# The effects of oral medroxyprogesterone acetate combined with conjugated equine estrogens on inflammation in postmenopausal women: a systematic review and meta-analysis of randomized controlled trials

**DOI:** 10.3389/fendo.2025.1643413

**Published:** 2025-10-15

**Authors:** Jiahui Qiu, Yunan He, Jinhong Li, Mohammad Safargar, Kousalya Prabahar, Qingquan Shi

**Affiliations:** ^1^ Center of Reproductive Medicine, West China Second University Hospital, Sichuan University, Chengdu, Sichuan, China; ^2^ Key Laboratory of Birth Defects and Related Diseases of Women and Children, Sichuan University, Ministry of Education, Chengdu, Sichuan, China; ^3^ Student Research Committee, Tabriz University of Medical Sciences, Tabriz, Iran; ^4^ Department of Pharmacy Practice, Faculty of Pharmacy, University of Tabuk, Tabuk, Saudi Arabia

**Keywords:** medroxyprogesterone acetate, conjugated equine estrogens, inflammation, postmenopausal women, meta-analysis, hormone therapy, CRP, fibrinogen

## Abstract

**Background and Aim:**

Menopausal hormone therapy (MHT) remains a pivotal approach in managing menopausal symptoms; however, its effects on inflammation and cardiovascular risk markers are still under debate. In particular, the combination of medroxyprogesterone acetate (MPA) and conjugated equine estrogens (CEE) has shown variable impacts on inflammatory biomarkers. This systematic review and meta-analysis aimed to synthesize evidence from randomized controlled trials (RCTs) assessing the effects of oral MPA combined with CEE (MPA/CEE) on systemic inflammation in postmenopausal women.

**Methods:**

Thirteen RCTs (comprising 16 arms) reporting data on inflammatory markers, including C-reactive protein (CRP), fibrinogen, homocysteine, and interleukin-6 (IL-6), were included, with a total sample size of 2,278 participants. A random-effects model was used to calculate pooled weighted mean differences (WMDs) with 95% confidence intervals. Subgroup and sensitivity analyses were performed to explore heterogeneity, and publication bias was assessed using Egger's test and trim-and-fill methods.

**Results:**

MPA/CEE treatment was associated with a significant decrease in CRP levels (WMD = -0.173 mg/dL; 95% CI: -0.25 to -0.10; P < 0.001), particularly among postmenopausal women aged <60 years, trials with MPA doses ≤2.5 mg/day, and those with BMI <25 kg/m². In addition, a significant reduction in fibrinogen levels was observed (WMD = -60.588 mg/dL; 95% CI: -71.436 to -49.741; P < 0.001), especially at MPA doses ≤2.5 mg/day and in women with BMI <25 kg/m². No statistically significant changes were found in homocysteine or IL-6 levels.

**Conclusion:**

While MPA/CEE therapy significantly reduces CRP and fibrinogen, key inflammatory and cardiovascular risk markers, these findings suggest a notable protective effect of oral MPA/CEE on inflammation, highlighting the need for individualized therapeutic strategies based on patient risk profiles.

## Introduction

The menopausal transition is characterized by a decline in endogenous estrogen production, which can result in a range of undesirable symptoms, including vasomotor disturbances, vulvovaginal dryness and atrophy, decreased bone mineral density, and adverse changes in lipid metabolism (1). Beyond these clinical manifestations, menopause has also been associated with an increase in systemic inflammation, independent of chronological aging (2). Menopausal hormone therapy (MHT), administered as either estrogen alone or in combination with a progestin, has long been employed to relieve these symptoms and improve quality of life in postmenopausal women (3). Current guidelines from global organizations, including the North American Menopause Society and the International Menopause Society, recommend MHT as the most effective treatment for vasomotor symptoms and vulvovaginal atrophy in appropriately selected postmenopausal women, with an emphasis on individualized risk-benefit assessment (4, 5).

However, estrogen therapy has been shown to elevate circulating levels of C-reactive protein (CRP), a well-established marker of systemic inflammation (6). This finding is of particular clinical relevance, as elevated CRP levels are strongly linked to an increased risk of cardiovascular events (7). Randomized controlled trials (RCTs) evaluating the use of hormone replacement therapy (HRT) for cardiovascular disease prevention have, unexpectedly, revealed a rise in both venous and arterial thrombotic events following initiation of treatment (8). It remains unclear whether the increase in CRP reflects a generalized pro-inflammatory response mediated by upstream cytokines like interleukin-6 (IL-6), or whether alternative pathways are involved. For example, findings from the Postmenopausal Estrogen/Progestin Interventions (PEPI) trial indicated that while CRP levels rose during HRT, there were no corresponding increases in fibrinogen, E-selectin, or other acute-phase reactants (9). Although CRP elevation has been proposed as a possible mediator of HRT-associated risks, definitive clinical outcome data supporting this link are lacking. Some evidence suggests that estrogen therapy may provoke or exacerbate inflammation, potentially accelerating the development of atherosclerosis and thrombosis in women with predisposing risk factors (10). However, the inflammatory potential of unopposed estrogen remains a subject of debate (11). The relative contributions of estrogen versus progestins to systemic inflammation remain incompletely understood, with conflicting data on whether estrogen or progestins are the predominant mediators of CRP elevation.

Medroxyprogesterone acetate (MPA) is commonly prescribed as a progestin alongside estrogen in women with an intact uterus, to reduce the risk of endometrial hyperplasia and cancer (12). Notably, androgens possess anti-inflammatory properties (13), and because synthetic progestins such as MPA exhibit androgenic activity, their concurrent use with estrogen may help counterbalance the pro-inflammatory effects of estrogen therapy (14).

Despite the widespread use of MHT and recognition of its benefits, there remains uncertainty regarding the differential effects of various progestin doses on systemic inflammation and cardiovascular risk markers. While estrogen's impact on inflammatory biomarkers like CRP has been extensively studied, the role of progestins, especially MPA, in modulating these effects at different doses remains inadequately characterized. Moreover, conflicting evidence exists about whether lower progestin doses may confer superior anti-inflammatory benefits compared to higher doses (15, 16). Addressing these gaps is critical to optimizing hormone therapy regimens to maximize therapeutic benefits while minimizing cardiovascular risks.

Therefore, this systematic review and meta-analysis aims to evaluate the dose-dependent effects of oral MPA combined with conjugated equine estrogens (MPA/CEE) on inflammatory biomarkers in postmenopausal women.

## Materials and methods

### Search strategy

Two independent researchers conducted a comprehensive literature search across Scopus, PubMed/MEDLINE, EMBASE, and Web of Science to identify peer-reviewed articles published in English through August 2025. The search aimed to locate studies evaluating the effects of MPA/CEE on inflammatory biomarkers in postmenopausal women. A combination of Medical Subject Headings (MeSH) and free-text keywords was used to maximize the sensitivity and specificity of the search. Full details of the search strategy are provided in **Supplementary Table 1**.

### Inclusion criteria and exclusion criteria

Publications were included in our systematic review and meta-analysis if they met all of the following criteria: Population (P), postmenopausal women; Intervention (I), treatment with MPACEE; Comparison (C), randomized controlled trials featuring a placebo or control group; and Outcomes (O), reported measurable inflammation markers, specifically mean and standard deviation (SD) values for CRP, fibrinogen, homocysteine, and IL-6 at both baseline and at the end of the intervention. In this review, we excluded studies that did not provide adequate outcome data, along with unpublished reports, correspondence, commentaries, narrative reviews, brief communications, meta-analyses, ecological studies, and research conducted on animals.

### Data extraction

Two researchers independently screened all relevant RCTs and carefully selected those eligible for the meta-analysis. Data extraction was also performed independently by both researchers using a standardized form within Microsoft Excel (Microsoft Corp, Redmond, WA, USA). Any disagreements were resolved by consensus with the primary author. The following information was systematically collected from each RCT and recorded in the standardized Excel template: number of participants per group, mean age of participants, first author's name, treatment duration, study location, publication year, study design, participants' health status, mean and standard deviation (SD) values for CRP, fibrinogen, homocysteine, and IL-6 before and after intervention, as well as the prescribed MPA/CEE dose.

### Quality assessment

Two independent assessors evaluated the quality of evidence in the selected studies using the Cochrane Collaboration's risk of bias tool. This assessment covered key domains including random sequence generation, allocation concealment, blinding of participants and personnel, blinding of outcome assessors, handling of incomplete outcome data, and selective outcome reporting. These criteria formed the foundation for judging the overall quality and reliability of the evidence presented in the RCTs (17).

### Statistical analysis

In this meta-analysis, statistical analyses were conducted using Stata version 15 (Stata Corp., College Station, TX, USA). Because the included RCTs varied in participant characteristics, baseline BMI, intervention dosages, and study durations, we anticipated genuine between-study heterogeneity. Therefore, we predefined the use of a random-effects model (DerSimonian and Laird method) to generate pooled estimates, as this approach accounts for both within-study and between-study variance. A fixed-effect model, which assumes a single true effect size, was considered less appropriate in this context. Nevertheless, to ensure the robustness of our pooled results, sensitivity analyses were conducted by systematically excluding each study arm one at a time and recalculating the overall combined effect size.

We utilized a random-effects model based on the DerSimonian and Laird method to calculate pooled estimates of the intervention's impact on inflammation. Weighted mean differences (WMDs) with 95% confidence intervals (CIs) were derived from the mean and standard deviation (SD) values of both the MPA/CEE and control groups. A p-value of less than 0.05 was considered statistically significant. For studies reporting outcomes as percent change from baseline, we converted percent change into post-intervention means using mean_post_=mean_pre_×(1+%Δ/100). When SD of the percent change or post values were not reported, we estimated post-intervention SD by proportional scaling of the baseline SD: SD_post_≈ SD_pre_×(mean_post_/mean_pre_). When SDs for change scores were unavailable, they were estimated using the formula:

SD_change = √[(SD_baseline² + SD_final²) – (2 × R × SD_baseline × SD_final)],

where R represents the correlation coefficient. To ensure consistency, outcome units were converted to mg/dL when reported differently. For data presented as standard errors, medians, interquartile ranges, or ranges, we applied Cochrane Collaboration's recommended formulas to convert them to means and SDs (18, 19).

We utilized Pearson's chi-squared test (χ²) and Higgins' I² statistics to assess statistical heterogeneity among RCT arms. A significance threshold of p < 0.10 was applied, and heterogeneity was classified as low (25–49%), moderate (50–74%), or high (≥75%) based on predefined criteria. To further investigate heterogeneity, subgroup analyses were performed considering intervention duration, baseline characteristics, baseline body mass index (BMI), health status, and daily MPA/CEE dose. Subgroup analyses were performed primarily for outcomes with substantial heterogeneity (I² ≥ 75%) in order to explore potential sources of variability. For parameters with low or moderate heterogeneity, such as homocysteine, subgroup analyses were not conducted because of the limited number of available studies (n = 4), which would have made stratified analyses underpowered and potentially unreliable.

Potential publication bias was evaluated using funnel plots and confirmed with Egger's test, with p-values below 0.1 indicating statistical significance (20). When publication bias was detected, we applied the trim-and-fill method to adjust the effect sizes accordingly (21).

## Results

### Study selection

**Figure 1:**
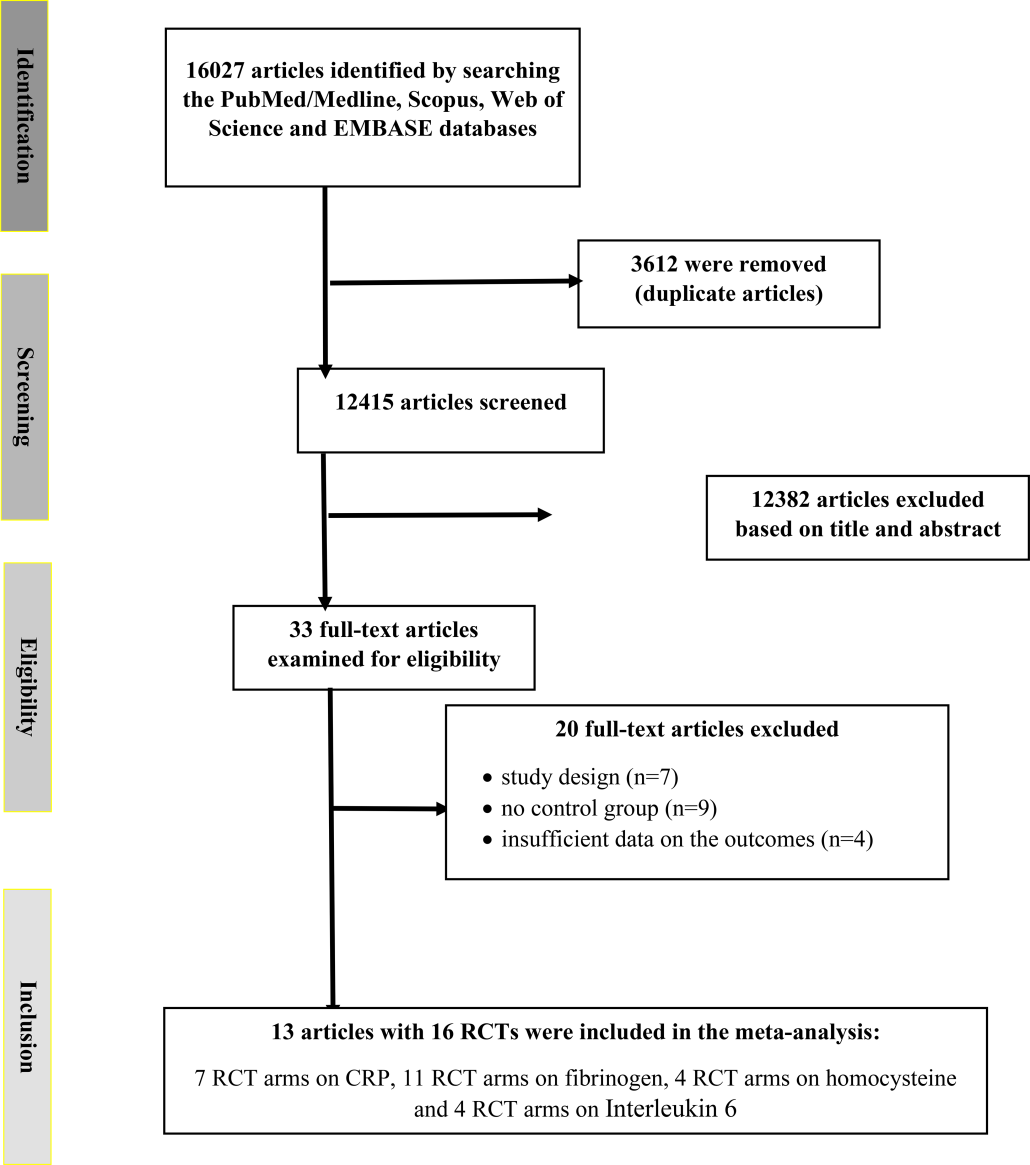
Flowchart depicting the study selection and inclusion process for the present meta analysis. RCT, randomized controlled trial(s).

We initially identified 16,027 publications across four databases. After removing duplicates, 12,415 unique records remained. A review of titles and abstracts led to the exclusion of 12,382 records, leaving 33 articles for full-text assessment. Of these, 20 were excluded based on eligibility criteria (**Figure 1**). Ultimately, 13 records with 16 arms were included in the final meta-analysis (22–34). These comprised 7 RCT arms reporting on CRP, 11 on fibrinogen, 4 on homocysteine, and 4 on IL-6.

### Characteristics of the included studies

The detailed characteristics of the included RCTs are presented in **Table 1**. The randomized controlled trials (RCTs) included in the analysis enrolled female participants and were conducted between 1997 and 2018. The duration of MPA/CEE treatment ranged from 3 to 36 months. The average age of participants spanned from 51 to 68.4 years, with a median age of 55.65 years. the daily dosage of MPA/CEE varied across studies, ranging from 1.5 mg to 10 mg for MPA and from 0.45 mg/day to 0.625 mg/day for CEE. These trials were conducted in several countries, including the United States, Denmark, Korea, Turkey, Finland, Japan, and Italy. Baseline body mass index (BMI) values ranged from 20.7 kg/m² to 32 kg/m².

**Table 1:** Characteristics of the eligible randomized controlled trials (RCTs) included in the meta-analysis.BMI, body mass index.

Author	Year	Country	Population	Participants' age (years)	Sample size: MAP/E2/placebo	Duration	Baseline BMI (kg/m^2^)	Outcome	MAP/E2 (mg/day)
Pickar, J. H.	2018	USA	Symptomatic postmenopausal women	54	35/36	3 months	24.8	Fibrinogen	Oral medroxyprogesterone acetate 2.5mg/day + conjugated equine estrogens 0.625mg/day
Skouby, S. O.	2015	Denmark	Healthy postmenopausal women	54.2	70/158	12 months	26.2	Fibrinogen	Oral medroxyprogesterone acetate 1.5mg/day + conjugated equine estrogens 0.45mg/day
Tuomikoski, P.	2010	Finland	Healthy symptomatic postmenopausal women	52.2	34/37	6 months	22.8	CRP	Oral medroxyprogesterone acetate 5 mg/day + oral estradiol valerate 2 mg/day
Rossouw, J. E.	2008	USA	Postmenopausal women	66.4	180/148	12 months	29.4	Fibrinogen, CRP, homocysteine, Interleukin 6	Oral medroxyprogesterone acetate 2.5mg/day + conjugated equine estrogens 0.625 mg
Spangler, L	2007	USA	Post-menopausal women experiencing vasomotor symptoms	52	27/73	3 months	32	Fibrinogen	Oral medroxyprogesterone acetate 2.5mg/day + conjugated equine estrogens 0.625 mg
Kooperbergl, C.	2007	USA	Postmenopausal women	68.4	249/178	12 months	28.3	CRP, homocysteine, Interleukin 6	Oral medroxyprogesterone acetate 2.5mg/day + conjugated equine estrogens 0.625mg
Sumino, H.(a)	2006	Japan	Postmenopausal women with hypertension	55.9	16/7	12 months	24.1	Fibrinogen, CRP	Oral medroxyprogesterone acetate 2.5mg/day for 12 days per month + conjugated equine estrogens 0.625mg/day
Sumino, H. (b)	2006	Japan	Postmenopausal women without hypertension	53.5	16/8	12 months	21.8	Fibrinogen	Oral medroxyprogesterone acetate 2.5mg/day for 12 days per month + transdermal 17b-estradiol 36 mcg/day
Sumino, H. (c)	2006	Japan	Postmenopausal women	54.8	28/13	12 months	22.7	CRP, Interleukin 6	Oral medroxyprogesterone acetate 2.5 mg/day + conjugated equine estrogens 0.625mg/day
Sumino, H. (d)	2006	Japan	Postmenopausal women	55.2	28/14	12 months	22.4	CRP, Interleukin 6	Oral medroxyprogesterone acetate 2.5mg/day + conjugated equine estrogens 0.625mg/day
Toprak, A	2005	Turkey	non-hysterectomies healthy postmenopausal women	51	20/15	3 months	27.6	homocysteine	Oral medroxyprogesterone acetate 2.5mg/day + conjugated equine estrogens 0.625mg/day
Osmanagaoglu, M. A.	2005	Turkey	Overweight or obese postmenopausal women	51	90/88	6 months	28	Fibrinogen	Oral medroxyprogesterone acetate 2.5mg/day + conjugated equine estrogens 0.625mg/day
Affinito, P.	2001	Italy	Postmenopausal women on maintenance hemodialysis	53.5	25/27	6 months	25.7	Fibrinogen	Oral medroxyprogesterone acetate 10 mg/day + transdermal estradiol 50 mg/day
Park, J. S.	2000	Korea	Postmenopausal women on maintenance hemodialysis	57	33/32	12 months	20.7	Fibrinogen, homocysteine	Oral medroxyprogesterone acetate 2.5mg/day + conjugated equine estrogens 0.625mg/day
Barrett-Connor, E. (a)	1997	USA	Healthy postmenopausal women	–	169/84	36 months	–	Fibrinogen	Oral CEE 0.625 daily + medroxyprogesterone acetate 10 mg
Barrett-Connor, E. (b)	1997	USA	Healthy postmenopausal women	–	169/85	36 months	–	Fibrinogen	Oral CEE 0.625 daily + medroxyprogesterone acetate 10 mg

Participants included a variety of postmenopausal populations: symptomatic and healthy postmenopausal women, those with hypertension, overweight or obese individuals, postmenopausal women with vasomotor symptoms, postmenopausal women without hypertension, non-hysterectomized healthy women, and those undergoing maintenance hemodialysis. The detailed characteristics of the included RCTs are presented in (**Table 1**). Risk of bias and methodological quality assessments are provided in (**Supplementary Table 2**).

### Findings from the meta-analysis

#### Effects of MPA/CEE treatment on C-reactive protein levels

**Figure 2:**
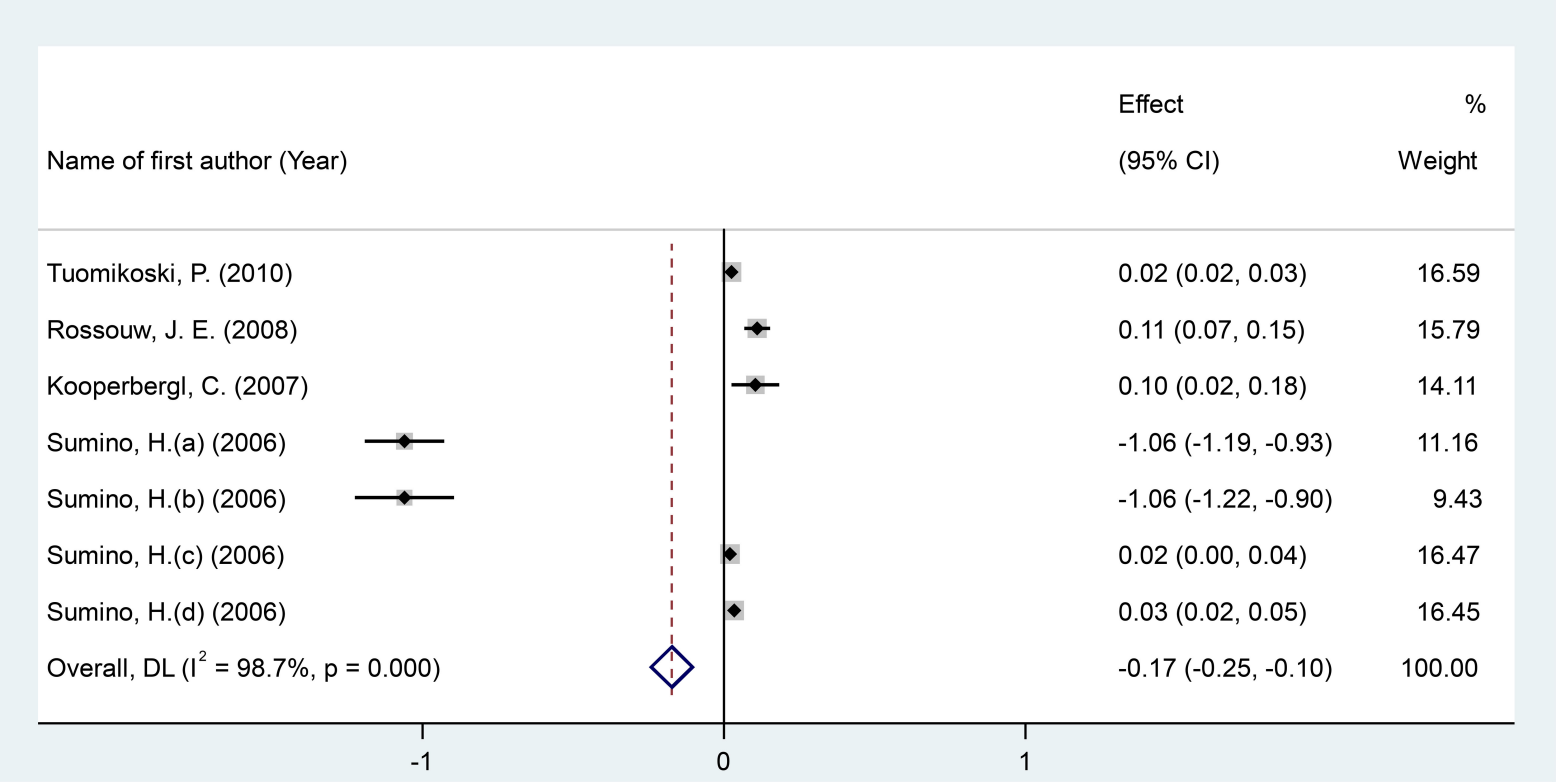
Forest investigating the effects of MAP/E2 on CRP. RCT, randomized controlled trial(s); WMD, Weighted mean difference; CI, confidence interval.

After pooling data from 7 RCT arms comprising a total of 998 participants (551 in the intervention group and 447 in the placebo group), we conducted a meta-analysis using a random-effects model. The results showed that MPA/CEE administration was associated with a significant decrease in CRP levels in postmenopausal women (WMD = -0.17 mg/dL; 95% CI: -0.25 to -0.10; P < 0.001) (**Figure 2**). A heterogeneity analysis indicated substantial variability among the included studies (I² = 98%, P < 0.001). Subgroup analyses revealed a significant decrease in CRP concentrations when MPA/CEE was administered at doses ≤2.5 mg/day (WMD = -0.26 mg/dL; 95% CI: -0.40 to -0.13; P< 0.001) compare to >2.5 mg/day (WMD = 0.02 mg/dL; 95% CI: 0.02 to 0.03; P< 0.001). The decrease was also more pronounced among participants aged <60 years (WMD = -0.29 mg/dL; 95% CI: -0.39 to -0.20; P< 0.001) compared to those aged ≥60 years (WMD = 0.10 mg/dL; 95% CI: 0.07 to 0.14; P< 0.001). Similarly, participants with a BMI <25 kg/m² showed a greater decrease in CRP (WMD = -0.29 mg/dL; 95% CI: -0.392= to -0.20; P< 0.001) compared to those with a BMI ≥25 kg/m² (WMD = 0.10 mg/dL; 95% CI: 0.071to 0.14; P< 0.001) (**Supplementary Figure 1**).

#### Effects of MPA/CEE treatment on fibrinogen levels

**Figure 3:**
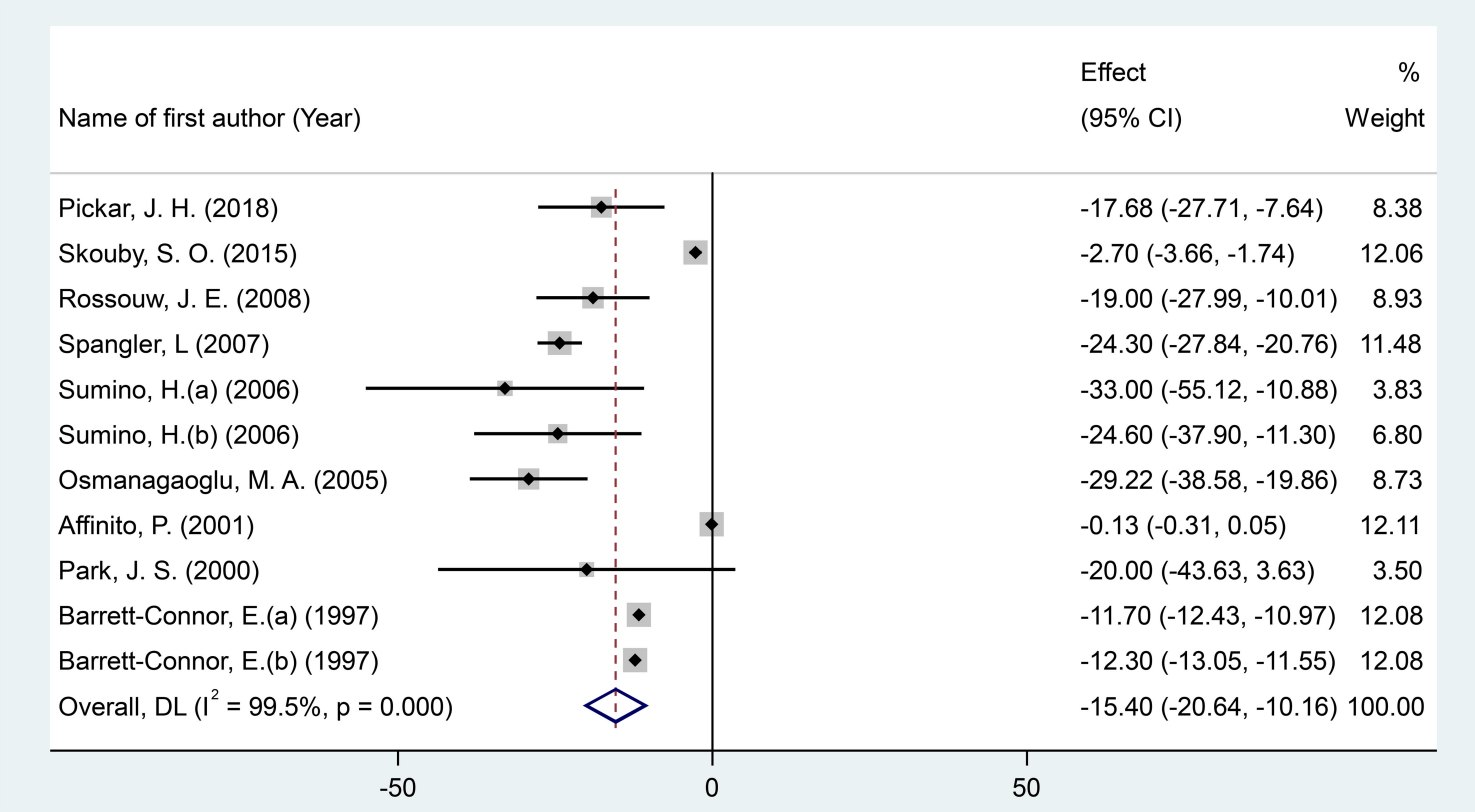
Forest plot of RCTs investigating the effects of MAP/E2 on Homocystteine. RCT, randomized controlled trial(s); WMD, Weighted mean difference; CI, confidence interval.

After pooling effect sizes from 11 RCT arms involving a total of 1,760 participants (830 in the intervention group and 930 in the placebo group), a random-effects meta-analysis revealed a significant reduction in fibrinogen levels following MPA/CEE treatment (WMD = -15.40 mg/dL; 95% CI: -20.64 to -10.15; P < 0.001) in postmenopausal women (**Figure 3**). However, there was considerable heterogeneity across studies (I² = 99.5%, P < 0.001).

Subgroup analyses showed significant decreases in fibrinogen concentrations with MPA/CEE doses ≤2.5 mg/day (WMD = -18.39 mg/dL; 95% CI: -24.35 to -12.44; P< 0.001), and in participants aged ≥60 years (WMD = -19 mg/dL; 95% CI: -27.99 to -10; P< 0.001) compared to those under 60 years (WMD = -14.77 mg/dL; 95% CI: -19.70 to -9.84; P< 0.001). Notable reductions were also observed in studies with treatment durations of ≤12 months (WMD = -17.59 mg/dL; 95% CI: -34.62 to -0.57; P = 0.043) compared to >12 months (WMD = -12.79 mg/dL; 95% CI: -17.49 to -8.08; P< 0.001), and in participants with a BMI <25 kg/m² (WMD = -21.52 mg/dL; 95% CI: -28.69 to -14.34; P< 0.001), compared to those with BMI ≥25 kg/m² (WMD = -12.87 mg/dL; 95% CI: -18.09 to -7.61; P< 0.001) (**Supplementary Figure 1**).

#### Effects of MPA/CEE treatment on homocysteine levels

**Figure 4:**
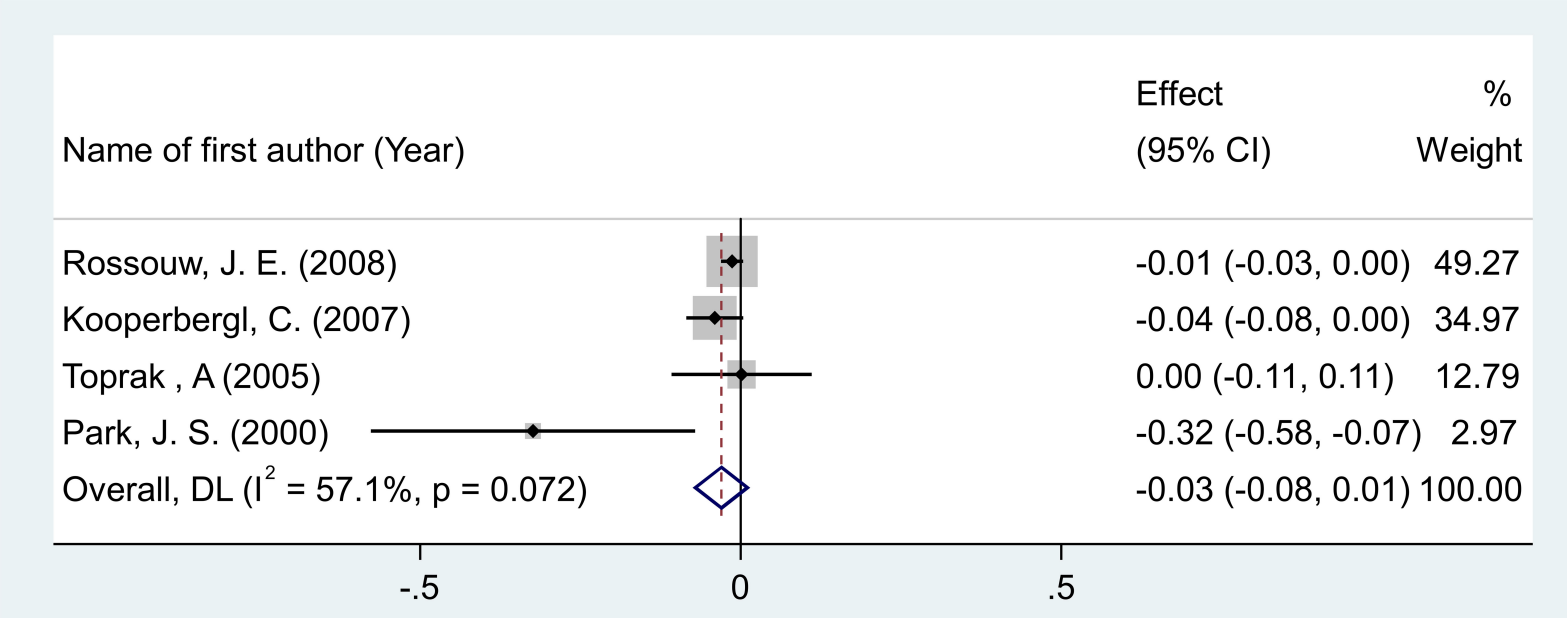
Forest plot of RCTs investigating the effects of MAP/E2 on Interleukin-6. RCT, randomized controlled trial(s); WMD, Weighted mean difference; CI, confidence interval.

After pooling effect sizes from 4 RCT arms, involving a total of 855 participants (482 in the intervention group and 373 in the placebo group), a random-effects meta-analysis found no significant reduction in homocysteine levels following MPA/CEE treatment (WMD = -0.03 mg/dL; 95% CI: -0.07 to 0.01; P = 0.186) in postmenopausal women (**Figure 4**). The analysis showed moderate heterogeneity among the studies, which was not statistically significant (I² = 57.1%, P = 0.072). Although moderate heterogeneity was observed for homocysteine (I² = 57.1%), subgroup analyses were not performed due to the small number of included RCTs, which would have limited the reliability of stratified results.

#### Effects of MPA/CEE treatment on interleukin-6 levels

**Figure 5:**
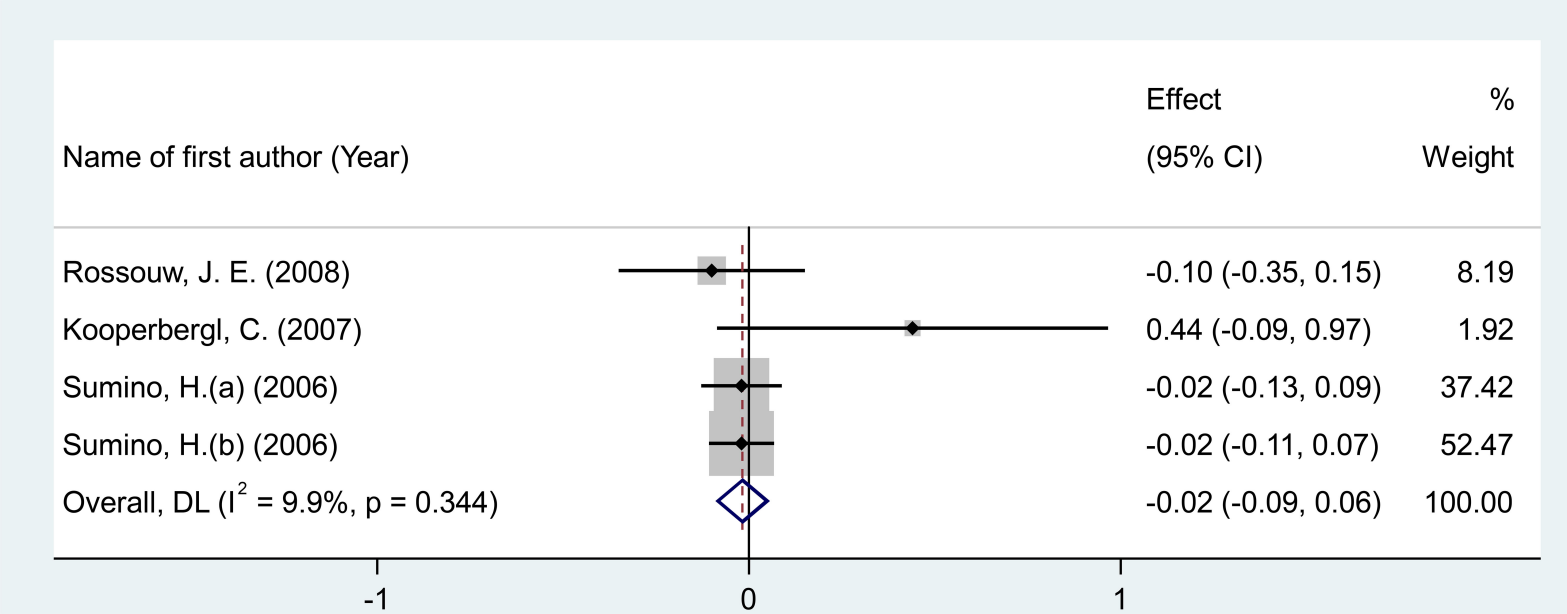
Forest plot of RCTs investigating the effects of MAP/E2 on fibrinogen. RCT, randomized controlled trial(s); WMD, Weighted mean difference; CI, confidence interval.

After pooling effect sizes from 4 RCT arms, involving a total of 865 participants (485 in the intervention group and 380 in the placebo group), a random-effects meta-analysis found no significant reduction in IL-6 concentrations following MPA/CEE treatment (WMD = -0.018 pg/mL; 95% CI: -0.09 to 0.05; P = 0.635) in postmenopausal women (**Figure 5**). The analysis revealed low and non-significant heterogeneity among the included studies (I² = 9.9%, P = 0.344).

### Sensitivity analysis and publication bias

We performed sensitivity analyses to assess the robustness of the overall findings by systematically excluding each RCT arm one at a time and recalculating the pooled effect sizes. None of these exclusions significantly impacted the overall results (**Supplementary Figure 2**). Additionally, no evidence of publication bias was detected for the pooled effect sizes of CRP, homocysteine, and IL-6 levels, as indicated by visual inspection of the funnel plots. These findings were further supported by Egger's test results (**Supplementary Figure 3**). However, we found significant publication bias for fibrinogen (P = 0.053); yet, the trim-and-fill test did not identify any potentially missing (unpublished) studies.

## Discussions

This comprehensive meta-analysis of RCTs aimed to consolidate all available evidence on the effects of MPA/CEE on inflammation-related biomarkers in postmenopausal women. The analysis included data from 16 studies, covering 7 trial arms on CRP, 11 on fibrinogen, 4 on homocysteine, and 4 on IL-6, based on clearly defined inclusion and exclusion criteria. The primary focus was on postmenopausal women, encompassing both healthy individuals and those with existing health conditions.

A key finding of this review was the significant reduction in fibrinogen levels following MPA/CEE administration. Fibrinogen is a critical glycoprotein involved in coagulation and inflammatory processes, including macrophage adhesion and cytokine production (35). Beyond its role in acute inflammation, elevated fibrinogen contributes to chronic low-grade inflammation (36), serving as a reliable biomarker for systemic inflammation and an established predictor of cardiovascular disease risk (37). Subgroup analyses revealed that the reduction in fibrinogen was particularly significant in women aged ≥60 years and among those receiving MPA/CEE doses of ≤2.5 mg/day. These findings align with previous RCTs showing favorable changes in fibrinogen with lower MPA/CEE doses (38). Since fibrinogen levels tend to rise with age, the pronounced effect in older women may be attributed to higher baseline concentrations (39). Indeed, elevated plasma fibrinogen has been associated with a 15% increased risk of cardiovascular disease, underscoring its clinical relevance as a risk stratification tool (40). While our results suggest that MPA/CEE may mitigate inflammation via reductions in fibrinogen, it is important to recognize that biomarkers offer only indirect insights into actual cardiovascular outcomes. Therefore, large-scale prospective studies are needed to further explore this association.

MPA/CEE use was associated with a modest but statistically significant decrease in CRP levels (WMD = -0.16 mg/dL). However, no significant effects were observed for homocysteine (WMD = -0.03 mg/dL) or IL-6 (WMD = -0.018 pg/mL). These findings corroborate earlier reports that estrogen therapy, either alone or combined with a progestin, can decrease CRP levels (41). However, the mechanism behind the stimulation of CRP is debated; various studies report differing outcomes regarding the impact of estrogen, with or without progestins, on IL-6 (41). Moreover, subgroup analyses revealed a significant decrease in CRP concentrations when MPA/CEE was administered at doses ≤2.5 mg/day (WMD = -0.26 mg/dL). One possible explanation for the greater reductions in CRP and fibrinogen at lower progestin doses relates to the nuanced immunomodulatory effects of progestins. Lower doses may more effectively balance the suppression of pro-inflammatory cytokines and inhibition of nuclear factor-kappa B activity, thereby reducing inflammation without inducing receptor desensitization or counter-regulatory mechanisms that could blunt anti-inflammatory responses at higher doses (42, 43). This dose-dependent effect has been observed in studies of MPA and other progestins, suggesting that minimal effective dosing may optimize anti-inflammatory benefits.

The underlying mechanisms driving CRP declined remain unclear, especially given inconsistent data on the role of IL-6 in this process. Notably, findings from the PEPI study revealed that changes in IL-6 and CRP were positively correlated in progestin-treated groups, but negatively correlated in those receiving estrogen alone. This suggests that progestins may amplify IL-6-mediated CRP production, while alternative pathways may be responsible in estrogen-only regimens (44). These results support the hypothesis that progestins, rather than oral estrogens, are the primary contributors to CRP elevation through inflammatory signaling in the context of combined hormone therapy. Interestingly, no meaningful differences were observed based on the type of progestin or regimen (cyclic vs. continuous). While these findings provide important insights into the inflammatory signaling of combined hormone therapy, they do not originate from the present analysis.

Our results build upon this framework by demonstrating dose-dependent effects of MPA/CEE on inflammatory biomarkers, suggesting that progestin dosing may be a critical factor in modulating systemic inflammation. This distinction may partly explain the conflicting evidence regarding estrogen's role in CRP elevation described in the introduction. Whereas estrogen alone may elevate CRP through hepatic metabolic pathways unrelated to classical inflammatory cytokines, the progestin component in combined therapy appears to be a more direct contributor to inflammatory signaling and CRP production (45).

Therefore, rather than contradicting earlier observations, our findings refine the understanding of hormone therapy's inflammatory effects by highlighting the complex and dose-dependent interplay between estrogen and progestins. Further mechanistic studies are needed to clarify these pathways and optimize therapeutic strategies.

Importantly, our subgroup analyses revealed that MPA/CEE therapy at higher progestin doses, in older women, and in those with higher BMI was associated with significant increases in CRP levels, indicating a possible pro-inflammatory response under these conditions. This contrasts with the overall CRP reductions observed at lower doses and in younger or leaner subgroups, underscoring a complex interplay between hormone dose, patient characteristics, and inflammatory outcomes. The molecular mechanisms underlying this dose- and context-dependent pro-inflammatory effect remain to be fully elucidated. Potential pathways may involve enhanced activation of pro-inflammatory cytokines and nuclear factor-kappa B signaling at higher MPA doses, as well as synergistic effects with adipose tissue–derived inflammatory mediators in individuals with elevated BMI (46). Moreover, age-related changes in immune function and hormone metabolism could amplify these responses, increasing systemic inflammation and cardiovascular risk. Further research is needed to dissect these mechanisms, including studies exploring gene expression profiles, cytokine networks, and receptor-mediated effects of MPA and estrogen in diverse patient populations.

It is worth noting that while changes in homocysteine and IL-6 were not statistically significant, the decreases in fibrinogen and CRP were of a magnitude considered potentially meaningful in reducing inflammatory burden.

### Clinical implications

From a clinical perspective, MPA/CEE may be a favorable option for postmenopausal women with a lower risk of inflammation. However, individualized treatment remains essential. Clinicians should consider the total daily dose of MPA/CEE, along with patient-specific risk factors, when prescribing this therapy, particularly for those susceptible to inflammation-related complications.

### Strengths and limitations

A major strength of this systematic review and meta-analysis is that it represents the first comprehensive synthesis of RCT data evaluating the impact of MPA/CEE on inflammatory biomarkers in women.

Nonetheless, this study is not without limitations. Considerable heterogeneity was observed among the included trials, which may be attributed to variations in treatment duration, study populations, and demographic characteristics. Additionally, the methodological quality of some trials raised concerns, necessitating cautious interpretation of the results. The limited number of studies evaluating homocysteine and IL-6 (only four arms each) restricts the generalizability of findings for these specific markers. Furthermore, because fewer than 10 studies were available for some biomarkers, the assessment of publication bias using funnel plots, Egger's test, or trim-and-fill was unreliable, and these results should therefore be interpreted with caution.

## Conclusion

This systematic review and meta-analysis demonstrates that MPA/CEE therapy significantly reduces fibrinogen and CRP levels, indicating a potential anti-inflammatory effect. As such, MPA/CEE could be considered for postmenopausal women at reduced risk of inflammation. However, due to the modest effects on other biomarkers and the inherent limitations of using surrogate markers like fibrinogen, clinical decision-making should be personalized. Future large-scale epidemiological studies are needed to confirm the long-term cardiovascular benefits of MPA/CEE and to further elucidate its role in modulating inflammation.

## Data Availability

The raw data supporting the conclusions of this article will be made available by the authors, without undue reservation.
