# Supplementary material for: The effects of oral medroxyprogesterone acetate combined with conjugated equine estrogens on inflammation in postmenopausal women: a systematic review and meta-analysis of randomized controlled trials
Source: Front Endocrinol (Lausanne). 2025 Oct 15;16:1643413. doi: 10.3389/fendo.2025.1643413 (PMC12568419; doi:10.3389/fendo.2025.1643413)
Supplement: Supplementary file 1 [file DataSheet1.docx]

**Supplementary Figure 1. subgroup analyses**

RCT, randomized controlled trial(s). WMD, weighted mean difference. CI, confidence interval. BMI, body mass index.

**MAP/E2 dosage (mg/day)**

**AGE**

**Length of intervention (months)**

**Baseline BMI (kg/m^2^)**
