# Supplementary material for: The effects of oral medroxyprogesterone acetate combined with conjugated equine estrogens on inflammation in postmenopausal women: a systematic review and meta-analysis of randomized controlled trials
Source: Front Endocrinol (Lausanne). 2025 Oct 15;16:1643413. doi: 10.3389/fendo.2025.1643413 (PMC12568419; doi:10.3389/fendo.2025.1643413)
Supplement: Supplementary file 2 [file DataSheet2.docx]

**Supplementary Figure 2.** Sensitivity analysis**.**

CI, confidence interval.

A) CRP

B) Fibrinogen

C) Homocysteine

D) Interleukin-6
