# Supplementary material for: The effects of oral medroxyprogesterone acetate combined with conjugated equine estrogens on inflammation in postmenopausal women: a systematic review and meta-analysis of randomized controlled trials
Source: Front Endocrinol (Lausanne). 2025 Oct 15;16:1643413. doi: 10.3389/fendo.2025.1643413 (PMC12568419; doi:10.3389/fendo.2025.1643413)
Supplement: Supplementary file 3 [file Table1.docx]

Supplementary Table 1. Search strategy.

| PubMed/MEDLINE |
| --- |
| ((((Medroxyprogesterone Acetate [MeSH Terms] OR Medroxyprogesterone Acetate [tiab] OR "Depo-Medroxyprogesterone Acetate" [tiab] OR " Depo Medroxyprogesterone Acetate" [tiab] OR "Provera" [tiab] OR "Sayana" [tiab] OR estrogen [Mesh] OR estrogen [tiab] OR estrogen replacement therapy [Mesh] OR estrogen replacement therapy [tiab] OR hormone replacement therapy [Mesh] OR hormone replacement therapy [tiab] OR estradiol [Mesh] OR estradiol [tiab] OR progestin therapy [Mesh] OR progestin therapy [tiab] OR progestin [Mesh] OR progestin [tiab] OR *progesterone [Mesh] OR *progesterone [tiab] OR HRT [Mesh] OR HRT [tiab] OR medrogestone [Mesh] OR medrogestone [tiab])) AND ((((("Clinical Trials as Topic"[Mesh] OR "Cross-Over Studies"[Mesh] OR "Double-Blind Method"[Mesh] OR "Single-Blind Method"[Mesh] OR "Random Allocation"[Mesh] OR RCT[Title/Abstract] OR "Clinical Trial" [Publication Type] OR "Controlled Clinical Trials as Topic"[Mesh] OR "Intervention Studies"[Title/Abstract] OR "intervention"[Title/Abstract] OR Trial[Title/Abstract] OR "controlled trial"[Title/Abstract] OR "randomized"[Title/Abstract] OR "randomised"[Title/Abstract] OR "random"[Title/Abstract] OR "randomly"[Title/Abstract] OR "placebo"[Title/Abstract] OR "assignment"[Title/Abstract]))))) |
