# Supplementary material for: The effects of oral medroxyprogesterone acetate combined with conjugated equine estrogens on inflammation in postmenopausal women: a systematic review and meta-analysis of randomized controlled trials
Source: Front Endocrinol (Lausanne). 2025 Oct 15;16:1643413. doi: 10.3389/fendo.2025.1643413 (PMC12568419; doi:10.3389/fendo.2025.1643413)
Supplement: Supplementary file 4 [file Table2.docx]

**Supplementary Table 2:** The risk of bias in studies included in this study

|  | Random Sequence Generation (selection bias) | Allocation concealment (selection bias) | Blinding of participants and personnel (performance bias) | Blinding of outcome assessment (detection bias) | Incomplete outcome data (attrition bias) | Selective reporting (reporting bias) | Other bias | AHRQ standards |
| --- | --- | --- | --- | --- | --- | --- | --- | --- |
| Pickar, J. H. |  |  |  |  |  |  |  | Good |
| Skouby, S. O. |  |  |  |  |  |  |  | Good |
| Tuomikoski, P. |  |  |  |  | 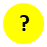 |  |  | Fair |
| Rossouw, J. E. |  |  |  |  |  |  |  | Good |
| Spangler, L |  |  |  |  |  |  |  | Good |
| Kooperbergl, C. |  |  |  |  |  | 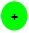 |  | Good |
| Sumino, H.(a) | 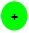 | 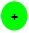 | 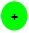 | 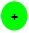 | 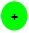 | 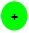 | 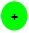 | Good |
| Sumino, H.(b) |  |  |  |  |  |  |  | Good |
| Sumino, H.(c) | 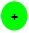 | 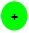 | 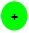 | 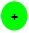 | 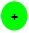 | 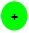 | 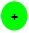 | Good |
| Sumino, H.(d) |  |  |  |  |  |  |  | Good |
| Toprak , A |  |  |  |  |  |  |  | Good |
| Osmanagaoglu, M. A. |  |  |  |  |  |  |  | Good |
| Affinito, P. |  | 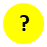 | 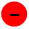 | 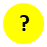 |  |  |  | Poor |
| Park, J. S. |  |  |  |  |  |  |  | Good |
| Barrett-Connor, E.(a) |  |  |  |  |  |  |  | Good |
| Barrett-Connor, E.(b) |  |  |  |  |  |  |  | Good |
